# Austenite grain growth simulation considering the solute-drag effect and pinning effect

**DOI:** 10.1080/14686996.2016.1244473

**Published:** 2017-01-23

**Authors:** Naoto Fujiyama, Toshinobu Nishibata, Akira Seki, Hiroyuki Hirata, Kazuhiro Kojima, Kazuhiro Ogawa

**Affiliations:** ^a^ Research & Development, Nippon Steel & Sumitomo Metal Corporation, Ltd, Futtsu, Japan; ^b^ Research & Development, Nippon Steel & Sumitomo Metal Corporation, Ltd, Amagasaki, Japan; ^c^ Nippon Steel & Sumikin Technology Co., Ltd., Amagasaki, Japan

**Keywords:** Austenite grain growth, HAZ, solute-drag effect, pinning effect, low carbon steel, phase-field method, 10 Engineering and structural materials, 400 Modeling / simulations, 106 Metallic materials, 100 Materials

## Abstract

The pinning effect is useful for restraining austenite grain growth in low alloy steel and improving heat affected zone toughness in welded joints. We propose a new calculation model for predicting austenite grain growth behavior. The model is mainly comprised of two theories: the solute-drag effect and the pinning effect of TiN precipitates. The calculation of the solute-drag effect is based on the hypothesis that the width of each austenite grain boundary is constant and that the element content maintains equilibrium segregation at the austenite grain boundaries. We used Hillert’s law under the assumption that the austenite grain boundary phase is a liquid so that we could estimate the equilibrium solute concentration at the austenite grain boundaries. The equilibrium solute concentration was calculated using the Thermo-Calc software. Pinning effect was estimated by Nishizawa’s equation. The calculated austenite grain growth at 1473–1673 K showed excellent correspondence with the experimental results.

## Graphical abstract

**Figure:**
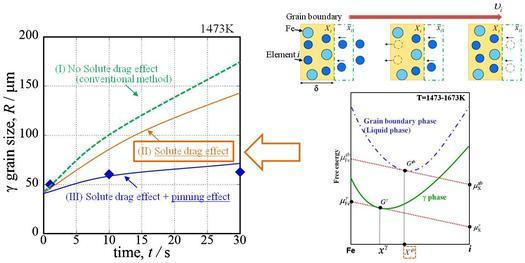

## 1. Introduction

High heat input welding is often employed in industrial fields such as ship building and civil engineering for low alloy steel structures. In high heat input welding, the heat affected zone (HAZ) adjacent to the fusion line is heated to nearly the melting point of steel by arc energy. Therefore, the austenite grain size in the area becomes coarse, and the toughness of the HAZ is generally lower than that of the base metal.[1–3] A fine HAZ microstructure is effective for obtaining high HAZ toughness, and some technical proposals, such as the pinning effect and intragranular ferrite (IGF), have been proposed in this regard. For example, oxides such as TiO,[4–6] MnAl_2_O_4_,[7, 8] Ti_2_O_3_,[9] Ti_2_O_3_-TiN-MnS,[10–12] Ti_2_O_3_-MnS-BN [13] and TiN-MnS [14] have an IGF nucleus, and inclusions such as TiN,[15–18] REM(O,S)-TiN,[19] oxide containing Ca and sulfide containing Mg [20] are reported as pinning particles. However, the optimum conditions of inclusions, including their size and distribution, for improving HAZ toughness are unclear. Furthermore, clarifying each factor’s influence on HAZ toughness through experiments is difficult. Therefore, it is important to develop numerical simulations for austenite grain growth. In previous research into simulating austenite grain growth, Burke proposed the following grain growth equation [21]:

(1) $$R^{n}-R_{0}^{n}=kt$$

where *R* is the radius of the grain (m), *t* is time (s), *k* is the kinetic constant (m^2^ s^−1^), and *n* is selected from 2 to 4 based on the rate-limiting steps. In addition, the grain growth is represented by the following kinetic equation for grain growth by Gibbs-Thomson effect:

(2) $$\frac{\text{d}R}{\text{d}t}=M^{gb}\times \frac{2\sigma V}{R}$$

where *M* ^gb^ is grain boundary mobility (m s^−1^ J^−1^ mol), *σ* is grain boundary energy (J m^−2^), and *V* is the molar volume (m^3^ mol^−1^). This equation represents the mobility and capillary effect, and it can be replaced with Burke’s equation. The solute-drag effect is also useful for restraining austenite grain growth in low alloy steel. This effect has a lower grain kinetic constant resulting from the segregation of impurities, and it has been researched by Lücke and Detert,[22] Cahn,[23] Hillert and Sundman,[24] and Purdy and Brechet.[25]

Although many researchers have discussed the solute-drag effect with mathematical models and experiments, most have been binary or ternary systems. These systems predict only the solute-drag effect, and no research has yet been conducted on how much influence this effect has on austenite grain growth.[26–28] This paper describes a new solute-drag effect prediction model for multi-element systems and applies it to an austenite grain growth simulation.

In addition to the solute-drag effect, the pinning effect suppresses austenite grain growth using inclusions. Zener proposed an equation for predicting the pinning effect [29]:

(3) $$R=\frac{4}{3}\frac{r}{t}$$

where *f* is the volume fraction of the inclusion and *r* is the radius of the inclusion (m). Based on Zener’s analysis, many researchers have studied the pinning effect.[30–35]

However, the pinning effect cannot be obtained by predicting the effect on austenite grain growth in combination with the solute-drag effect. This paper also describes a method for predicting austenite grain growth behavior that considers both the solute-drag and pinning effects.

## 2. Experimental procedure

### 2.1. Materials

The chemical compositions of the steels are listed in Table 1. A slab 100 mm thick, 180 mm wide, and 200 mm long was manufactured by vacuum melting followed by heating at 1423 K for 2 h and forging in the laboratory. Subsequently, the slab was heated at 1423 K for 2 h and rolled to 20 mm thick, 200 mm wide, and 800 mm long by hot rolling, after which it was air cooled to 1053 K and water cooled from 1023 K to 723 K.

**Table 1.** Chemical compositions of the steels (mass%).

Heat	C	Si	Mn	P	S	Ti	Nb	Al	N	O
Steel A	0.05	0.14	1.60	0.01	0.002	–	0.006	0.02	0.0050	0.002
Steel B	0.05	0.14	1.60	0.01	0.002	0.012	0.006	0.02	0.0038	0.002

### 2.2. Methods

A sample with a size of 11 × 11 × 60 mm was taken from the prepared steel plate and heated to simulate the HAZ (heating conditions: temperature increase of 100 K/s—1473–1673 K × 1–60 s—quench (He gas)). To measure the initial austenite grain size, the microstructure was observed using an optical microscope.

Furthermore, electrolytic extraction was performed using test pieces of steel B that had been given a simulated thermal cycle, and the residue was analyzed for Ti and N.

## 3. New austenite grain growth model

### 3.1. Equation of austenite grain growth considering the solute-drag effect

**Figure 1.**
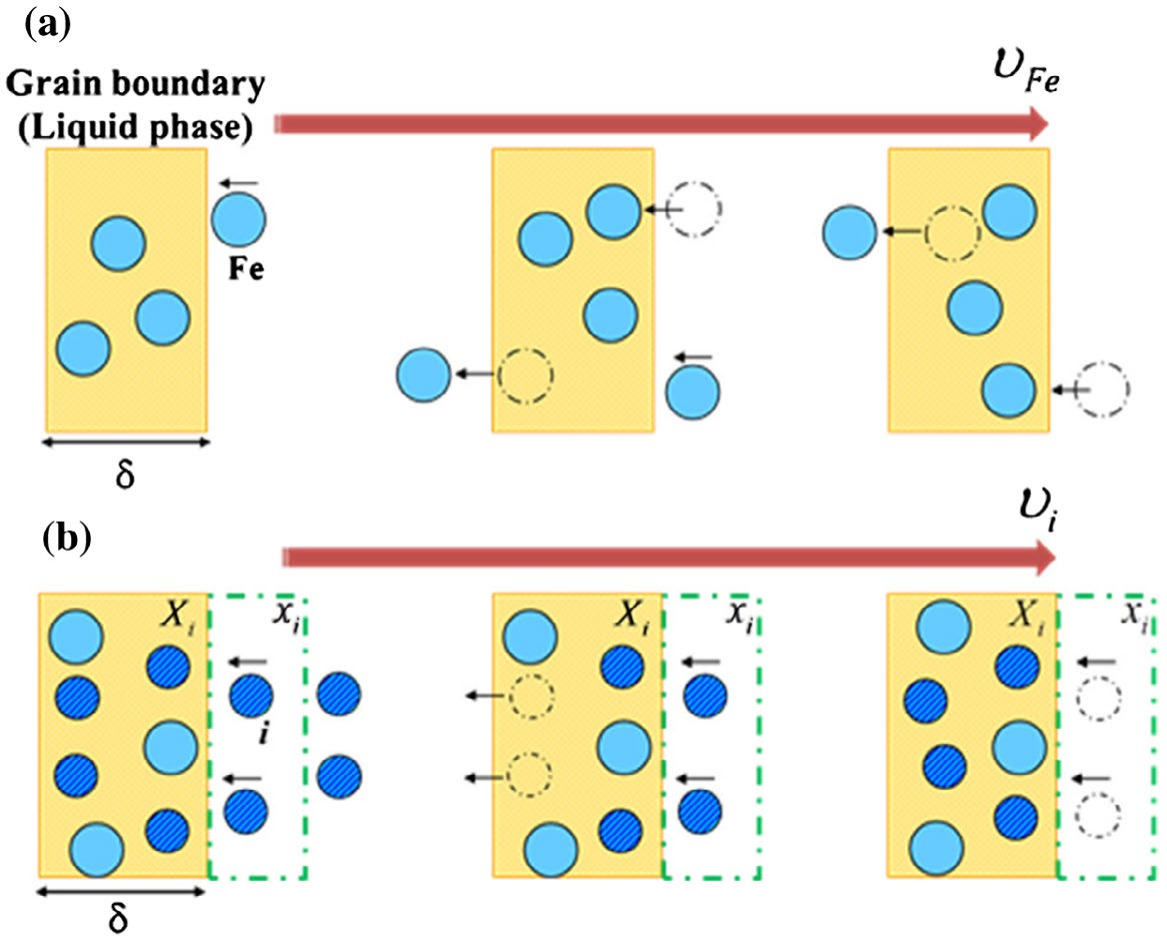
Schematics of grain boundary movement (a) in pure iron and (b) including solute.[16]

Figure 1 shows the schematics of grain boundary movement in (a) pure iron and (b) including solute.[36] This model assumes the grain boundary is liquid phase and the number of atoms existing in the grain boundary is constant. In Figure 1(a), when an iron atom enters the grain boundary, another iron atom must exit the grain boundary. Thus, the grain boundary moves through these entering and exiting atoms. This behavior is represented by Equation (4):

(4) $$\upsilon_{Fe}=I_{Fe^{\cdot }}\delta$$

where *υ*_*Fe*_ is the moving velocity of iron atom (m s^−1^), *I*_*Fe*_ is the transition frequency (s^−1^), and *δ* is the grain boundary width (m).

In Figure 1(b), when element *i* is a solute, the content of element *i* at the austenite grain boundaries is higher than inside the austenite grains. This gap in the content of element *i* lowers the austenite grain boundary mobility. In the static state, element *i* flux exits the grain boundary at the same time as element *i* flux enters the grain boundary. Therefore, this relationship is represented by Equation (5):

(5) $$\frac{X_{i}\times \left(\delta /\tau_{i}\right)}{V}=\frac{x_{i}\times \upsilon_{Fe}}{V}$$

where *X*_*i*_ is the austenite grain boundary concentration (mol), τ_*i*_ is the time of staying in the grain boundary, and *x*_*i*_ is the molar concentration of the solute (mol). Based on Equation (5), the transition frequency of element *i* is represented by Equation (6):

(6) $$I_{i}=\frac{1}{\tau_{i}}=\frac{x_{i}}{X_{i}}\times \frac{\upsilon_{Fe}}{\delta }=\frac{x_{i}}{X_{i}}\times I_{Fe}$$

From Equation (6), the moving velocity of element *i* is represented by Equation (7):

(7) $$\upsilon_{i}=I_{i}\delta =\frac{x_{i}}{X_{i}}\times I_{Fe}\delta$$

The driving force for grain growth is the summation of the effects of iron and element *i*:

(8) $$\Delta G=\frac{2\sigma V}{R}=\frac{X_{Fe}}{X_{Fe}+X_{i}}\times \frac{\upsilon }{M_{Fe}^{gb}}+\frac{X_{i}}{X_{Fe}+X_{i}}\times \frac{\upsilon }{M_{i}^{gb}}$$

where $M_{Fe}^{gb}$ is the grain boundary mobility of pure iron (m s^−1^ J^−1^ mol) and $M_{i}^{gb}$ is the grain boundary mobility of element *i* (m s^−1^ J^−1^ mol). The sum of the grain boundary concentration of iron and element *i* is 1, so Equation (8) can be replaced by Equation (9):

(9) $$\frac{2\sigma V}{R}=\frac{\upsilon }{M_{Fe}^{gb}}\times \left(1-X_{i}\right)+\frac{\upsilon }{M_{i}^{gb}}\times X_{i}=\left(\frac{1-X_{i}}{M_{Fe}^{gb}}+\frac{X_{i}}{M_{i}^{gb}}\right)\upsilon$$

This, in turn, leads to Equation (10):

(10) $$\frac{\text{d}R}{\text{d}t}=\upsilon =\frac{1}{\frac{\left(1-X_{i}\right)}{M_{Fe}^{gb}}+\frac{X_{i}}{M_{i}^{gb}}}\times \frac{2\sigma V}{R}$$

The grain boundary mobility of pure iron including solute element *i* is in proportion to the transportation frequency of pure iron including element *i*. Thus, the grain boundary mobility of pure iron including element *i* is:

(11) $$M_{i}^{gb}=\frac{x_{i}}{X_{i}}\times M_{Fe}^{gb}$$

From Equations (10) and (11), the kinetic equation of grain growth with solute in binary systems is represented by Equation (12):

(12) $$\frac{\text{d}R}{\text{d}t}=\frac{1}{\left(1-X_{{}_{i}}^{gb}\right)+\frac{{\left(X_{{}_{i}}^{gb}\right)}^{2}}{x_{i}}}M_{Fe}^{gb}\times \frac{2\sigma V}{R}$$

when expanding this model to a multi-element system, it is necessary to sum the effect of each solute as shown in Equation (13):

　(13) $$\frac{\text{d}R}{\text{d}t}=\frac{1}{\left(1-\sum_{i\neq \text{Fe}}X_{{}_{i}}^{}\right)+\sum_{i\neq \text{Fe}}\frac{{\left(X_{{}_{i}}\right)}^{2}}{x_{i}}}M_{Fe}^{gb}\times \frac{2\sigma V}{R}$$

Some of the grains grow while others shrink. Moreover, grain boundaries that are linked to the grain boundary of adjacent crystal grains are less likely to move than a single grain boundary. Equation (13) can be represented by Equation (14) by analyzing the steady state growth of the polycrystalline microstructure.[37]

(14) $$\frac{\text{d}R}{\text{d}t}=\frac{1}{4}\frac{1}{\left(1-\sum_{i\neq \text{Fe}}X_{{}_{i}}^{}\right)+\sum_{i\neq \text{Fe}}\frac{{\left(X_{{}_{i}}\right)}^{2}}{x_{i}}}M_{Fe}^{gb}\times \frac{2\sigma V}{R}$$

### 3.2. Method of calculating the content of element *i* at austenite boundaries

**Figure 2.**
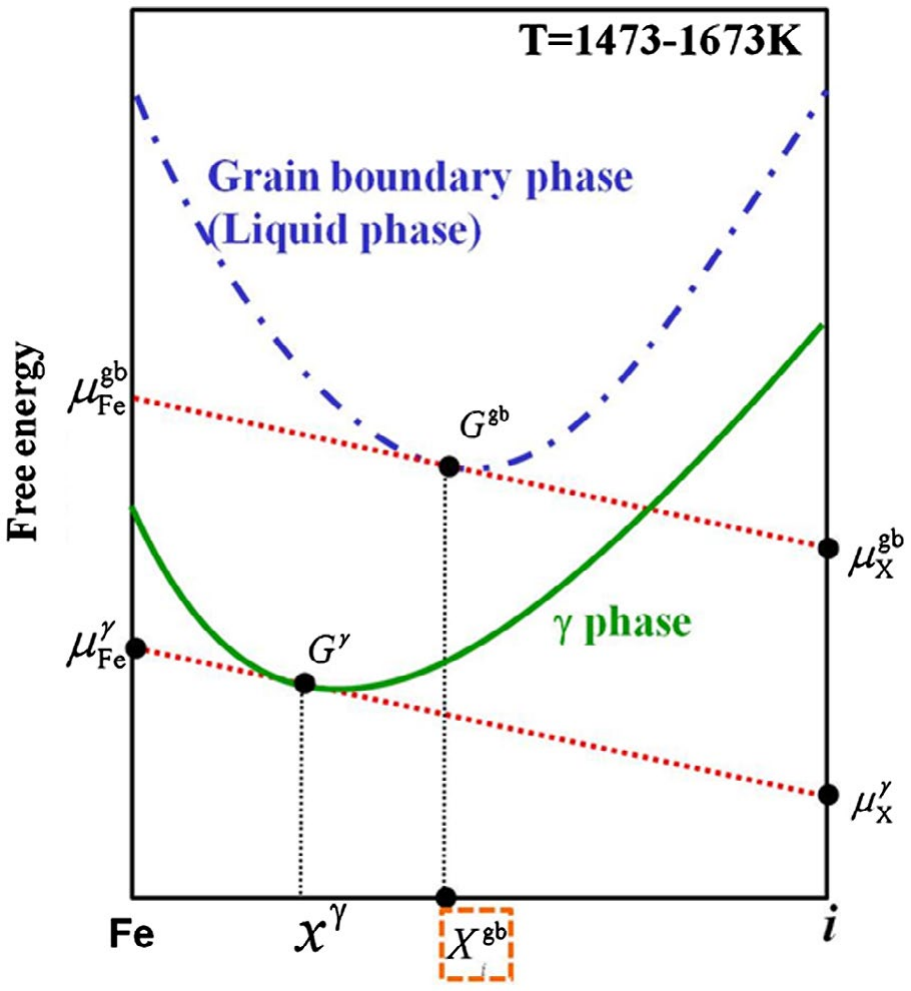
Schematic diagram of free energy curves.

From Equation (14), it is necessary to obtain the grain boundary concentration. Figure 2 shows a schematic diagram of free energy curves. $\mu_{Fe}^{gb}$ is the chemical potential of pure iron at the grain boundary, $\mu_{i}^{gb}$ is the chemical potential of element *i* at the grain boundary, $\mu_{Fe}^{\gamma }$ is the chemical potential of pure iron in the austenite, $\mu_{i}^{\gamma }$ is the chemical potential of element *i* in the austenite*,* $G^{gb}$ is the free energy at the grain boundary*,* $G^{\gamma }$ is the free energy in the austenite and $x_{i}^{\gamma }$ is the molar concentration of the solute in the austenite. We employed Hillert’s law [38] under the assumption that the austenite grain boundary is liquid phase. Thus, it is possible to estimate the equilibrium concentration of element *i* at the austenite grain boundaries from the intersection point between the grain boundary energy of the liquid phase and the slope of a line through $\mu_{Fe}^{gb}$ and $\mu_{i}^{gb}$. The calculations were performed using Thermo-Calc (http://www.thermocalc.com/products-services/software/thermo-calc/) and the TCFE6 database. In this research, C, Si, Mn, P, S, N and Nb were calculated as segregated elements, and it was possible to analyze the interaction among these elements using Thermo-Calc.

## 4. Results of an austenite grain simulation considering the solute-drag effect

### 4.1. Results of austenite grain growth observations

**Figure 3.**
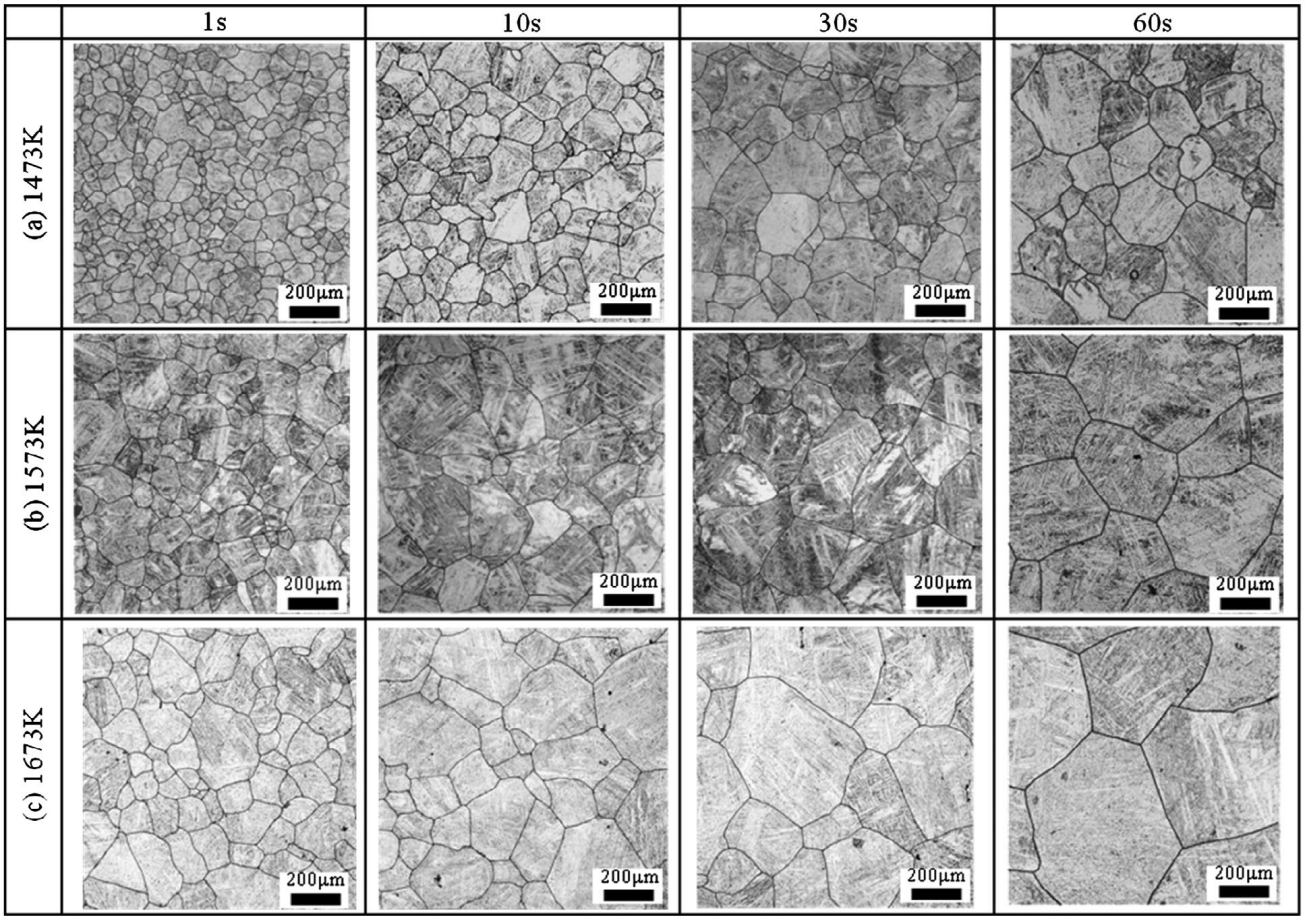
Microstructure of steel A at isothermal process

Figure 3 shows the microstructure of steel A in an isothermal heating process at (a) 1473 K, (b) 1573 K, and (c) 1673 K. As can be seen in Figure 3(a), the initial grain size increases with longer heating time. Also, as shown in Figure 3(b), the grain growth rate at 1573 K is higher than that at 1473 K, and this tendency becomes more drastic at 1673 K.

### 4.2. γ grain growth simulation considering the solute-drag effect

A calculation was conducted on the basis of the phase-field method [39, 40] using the phase-field equation [41]:

(15) $$\frac{\partial \phi_{i}}{\partial t}=M^{gb}\sum_{j=1}^{n}\frac{1}{v}\left\{\sum_{k=1}^{n}\left[\left(\sigma_{jk}-\sigma_{ik}\right)\left(\frac{\pi^{2}}{\delta^{2}}\phi_{k}+\nabla^{2}\phi_{k}\right)\right]+\frac{2\pi }{\delta }\sqrt{\phi_{i}\phi_{j}}\Delta G_{ij}\right\}$$

where ϕ_(*i*, *j*, *k*)_ is the phase field parameter, *ν* = 1–3 depending on balk and region of interface, σ_*jk*_ is the interface energy between grain *j* and *k*, σ_*ik*_ is the interface energy between grain *i* and *k*, *δ* is the interface width, and Δ*G*_*ij*_ is the driving force of grain growth between grain *i* and *j*. In this method, each grain is distinguished by an ID number used to track each grain growth and disappearance. Equation (15) includes the Gibbs–Tomson effect, which is the driving force of grain growth. Each ID has a phase-field parameter and the same mobility.

**Figure 4.**
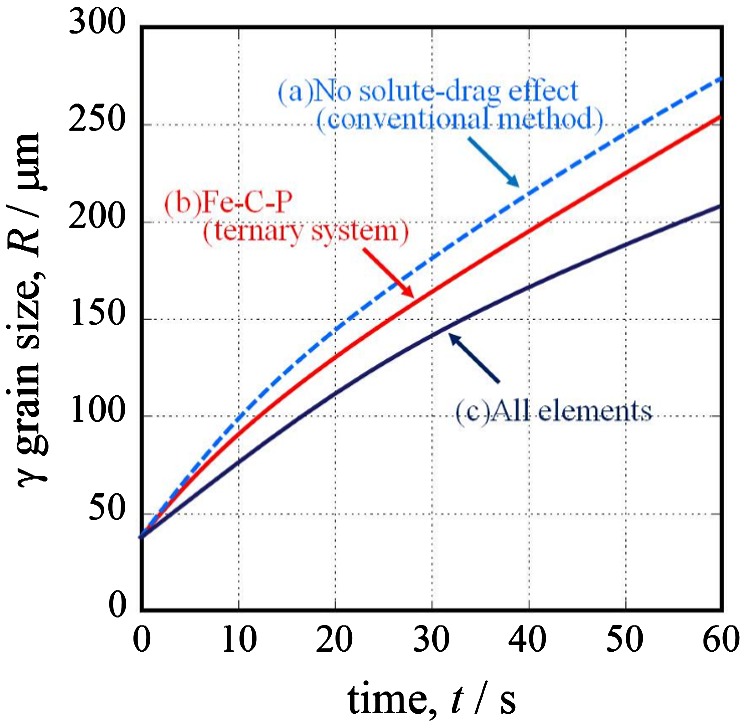
Results of the austenite grain growth simulation at 1473K (Steel A).

The calculation was performed using MICRESS^®^ [42] with a calculated area of 1000 × 1000 μm and interface width of 8 μm. In the phase field method, we used the proposed Equation (14) to input the grain boundary mobility. In this report, it is assumed that the grain boundary energy is constant (*σ* = 0.8 (J m^−2^)) because the effect of *X*_*i*_ is much stronger than that of the grain boundary energy. Figure 4 shows the result of the simulated austenite grain growth at 1473 K. The solid symbol plot is the prior austenite grain size from the experiment. When the grain size was calculated (a) without considering the solute-drag effect (conventional method [37]), the size differed from the experimental results. When the size was calculated with consideration for the solute-drag effect of (b) carbon and phosphorous, the calculated austenite grain size was smaller than the size without consideration for the solute-drag effect. Furthermore, when the calculation considered the solute-drag effect of (c) the multiple elements in Table 1, the calculated austenite grain size matched the experimental results. Austenite grain growth is restrained by adding elements. This means that the solute-drag effect becomes stronger as the number of solute elements increases.

**Figure 5.**
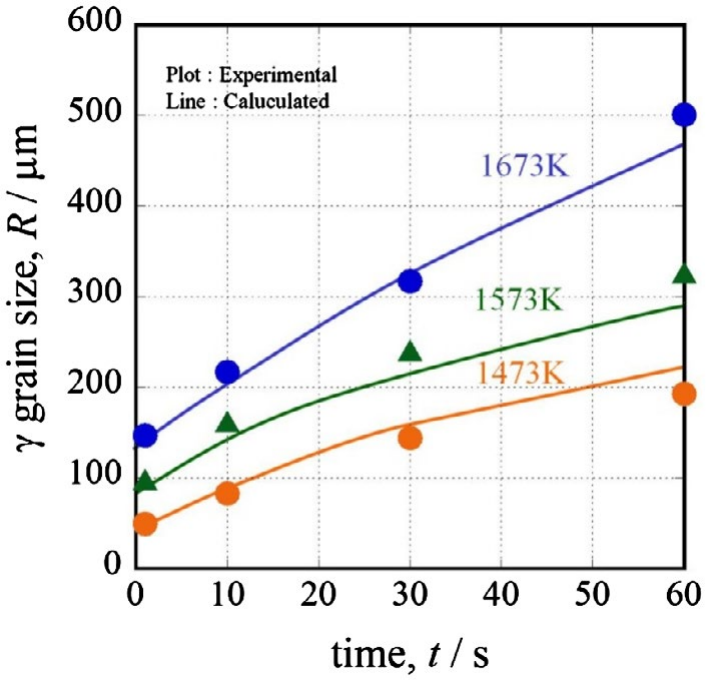
Results of austenite grain growth simulation considering the solute-drag effect at isothermal process (Steel A).

Figure 5 shows the results of the austenite grain growth simulation at 1473, 1573 and 1673 K. The grain growth rate increases as the temperature increases. The reason for this is that the solute-drag effect depends on temperature. The calculation shows sufficient correspondence to the experimental results. It is very important to consider the solute-drag effect when calculating the austenite grain growth behavior.

### 4.3. Results of the γ grain simulation considering the solute-drag effect and pinning effect

#### 4.3.1. Results of the γ grain growth observations

**Figure 6.**
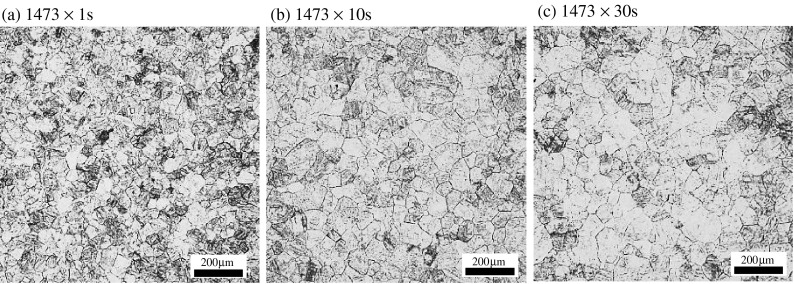
Microstructure of steel B (a–c) OM, (d) SEM.

**Figure 7.**
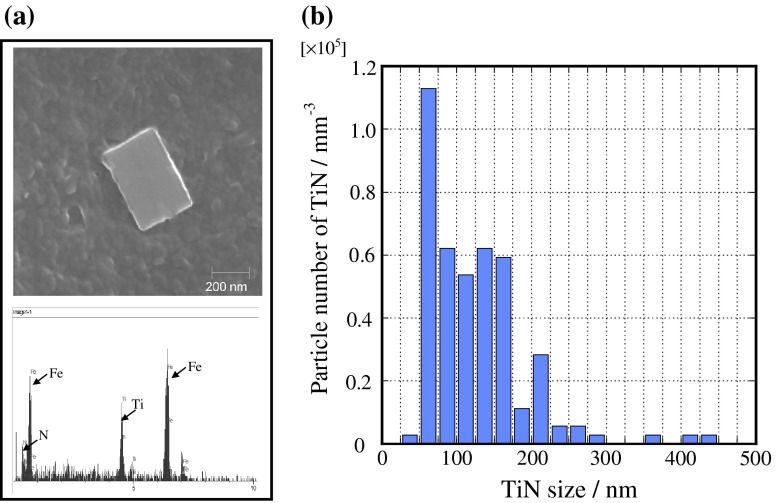
TiN distribution in Steel B.

Figure 6 shows the microstructures of steel B in an isothermal heating process at 1473 K. The initial grain size does not increase much with longer heating time. Figure 7 shows (a) Scanning Electron Microscope (SEM) images and the results of the EDX spectra of the pinning particle and (b) distribution of the pinning particle in steel B. The quantity of TiN was counted from the SEM images in the area of 1000 μm × 1000 μm. It can be seen that there is a lot of TiN in the steel. The most common TiN particle size is 50 nm. Therefore, the γ grain growth is restrained by the pinning effect of TiN.

#### 4.3.2. γ grain growth simulation considering the solute-drag effect and pinning effect

Equation (14) for the pinning effect:

(16) $$\frac{\text{d}R}{\text{d}t}=\frac{1}{4}\frac{1}{\left(1-\sum_{i\neq Fe}X_{i}^{gb}\right)+\sum_{i\neq Fe}\frac{X_{i}^{gb}}{x_{i}}}M_{Fe}^{gb}\times \left(\frac{\sigma V}{R}-\Delta G_{pin}\right)$$

where Δ*G*_*pin*_ is the pinning energy (J mol^−1^), which can be represented by Equation (17) [27, 28]:

(17) $$\Delta G_{pin}=\frac{3}{4}\frac{\sigma Vf^{2/3}}{r}$$

where *f* is the volume fraction of the inclusion and *r* is the radius of the inclusion (m). In MICRESS^®^, the pinning force *κ* needs to be input:

(18) $$\kappa =\frac{\Delta G_{pin}}{2\sigma V}$$

From Equation (17):

(19) $$\kappa =\frac{{3f}^{2/3}}{8r}$$

**Figure 8.**
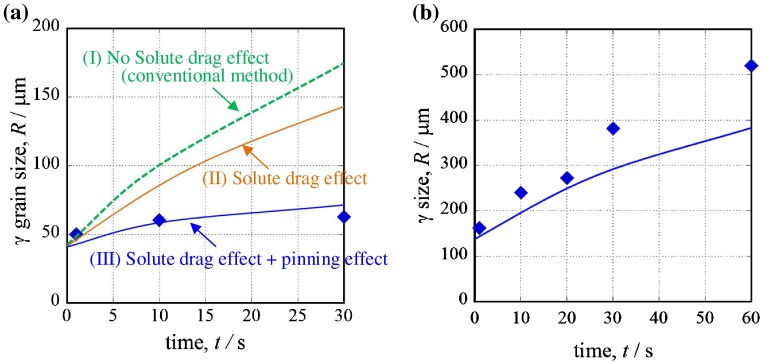
Results of the austenite grain growth simulation considering the solute-drag effect and pinning effect at (a)1473K, (b) 1673K.

Figure 8 shows the results of the austenite grain growth simulation of steel B that considered the pinning effect at (a) 1473 K and (b) 1673 K. Each solid symbol plot is the prior austenite grain size from the experiment, and the lines are the calculated results. We calculated the growth under three conditions: (I) without considering the solute-drag effect and pinning effect (conventional method [37]); (II) considering only the solute-drag effect; and (III) considering the solute-drag effect and pinning effect. In Figure 8(a), the volume fraction of TiN was calculated using Thermo-Calc, and a TiN radius of *r* = 50 nm, which is the most common size, was used. When the calculation does not consider the solute-drag and pinning effects, the calculated austenite grain size is much larger than the experimental results. When the calculation considers only the solute-drag effect, the calculated austenite grain size is smaller than the grain size calculated without considering the solute-drag and pinning effects, but it is still larger than the experimental results. When the calculation considers the solute-drag and pinning effects of TiN, the calculated size shows excellent correspondence with the experimental results. However, in Figure 8(b), the calculated austenite grain size does not show sufficient correspondence with the experimental results. TiN dissolved in the HAZ adjacent to the fusion line, and this reduced the pinning effect. The proposed equation cannot consider this, so the pinning effect is overestimated. Currently, we are trying to calculate austenite grain growth with consideration for this dissolution phenomenon.[43]

## 5. Conclusions

We proposed a new calculation model for predicting austenite grain growth behavior. This calculation model considers the solute-drag effect and pinning effect. The findings obtained are summarized as follows:

(1) The equation for austenite grain growth considering the solute-drag effect is based on the theory of grain boundary movement by transition of solute atoms. The solute-drag effect in this model can consider multi-element systems. To calculate the solute-drag effect, the grain boundary concentration of each element was obtained by Thermo-Calc under Hillert’s law. Simulation of the austenite grain growth in low alloy steel at 1473–1673 K by the phase-field method (MICRESS^®^) confirmed the validity of this approach.
(2) Austenite grain growth considering the pinning effect can calculate the grain growth by combining the proposed model and conventional equation of the pinning effect. The calculated result of austenite grain growth behavior for steel that has a TiN pinning effect at 1473 K shows sufficient correspondence to the experimental results. However, the calculated austenite grain size at 1673 K does not show sufficient correspondence with the experimental results because of TiN dissolution. In the future, we will attempt to calculate austenite grain growth considering this dissolution phenomenon.

## Disclosure statement

No potential conflict of interest was reported by the authors.
